# Getting topological photonics out of the laboratory

**DOI:** 10.1038/s41467-022-29492-1

**Published:** 2022-04-26

## Abstract

Bo Zhen (Assistant Professor, University of Pennsylvania), Andrea Blanco Redondo (Head of Silicon Photonics, Nokia Bell Labs), Alexander Szameit (Professor of Physics, University of Rostock), and Patrice Genevet (research scientist in photonics, Centre de recherche sur l’hétéro-épitaxie et ses applications, CNRS) talked to Nature Communications about the opportunities and challenges in the integration of topological photonics systems into real-world devices as well as envision new functionalities, but from a practical perspective.

1. Tell us about your research background and how it brought you to work on topological photonics?

**[Bo Zhen]** My journey started somewhere seemingly far from topology, at least at first glance. In 2013, we observed some new and interesting resonances in photonic crystal slabs. These resonances do not radiate any power to the environment and can have, in theory, infinitely long lifetimes. These resonances are now more well known as bound states in the continuum or BICs. What makes them even more interesting is their robust existence—we can change the geometric design by quite a bit, say 10% or 20%, yet we would still be able to find such states (BICs). It was then suggested (by Prof. Doug Stone) that we look into some sort of “topological protection” of these states—a rather exotic idea to us at that point—and it turned out to be very fruitful. Indeed, we found that BICs were fundamentally topological defects, which perfectly explains why they cannot radiate into the environment and why they always exist in such a robust manner. From then on, we were introduced to the field of topological photonics and have been having fun ever since.

**Figure:**
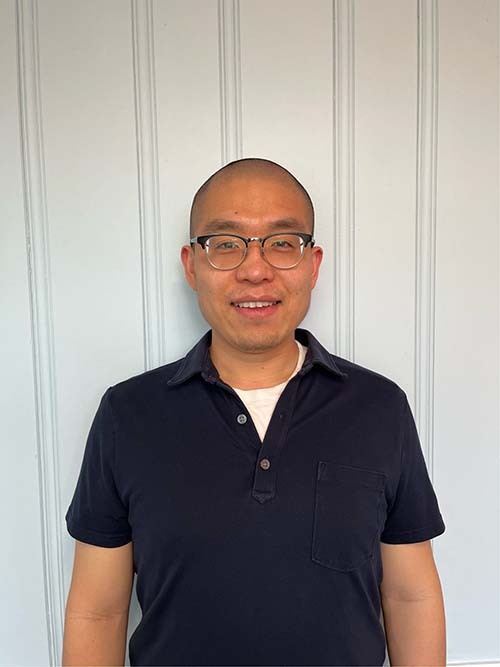
Bo Zhen

**[Andrea Blanco Redondo]** My research background is nonlinear integrated photonics and photonic bandgap materials, such as photonic crystals. Working with photonic crystals gave me an understanding of the dispersion relation and how to engineer periodic photonic platforms to create exotic dispersion profiles. Since the topology of photonics resides in the bands of their dispersion relation, working in topological photonics was a logical transition for me. In addition to this, my background in nonlinear optics inspired me to start leveraging topological photonics to protect quantum states of light, by exploring the nonlinear generation of entangled photons in topological platforms and studying their robustness.

**Figure:**
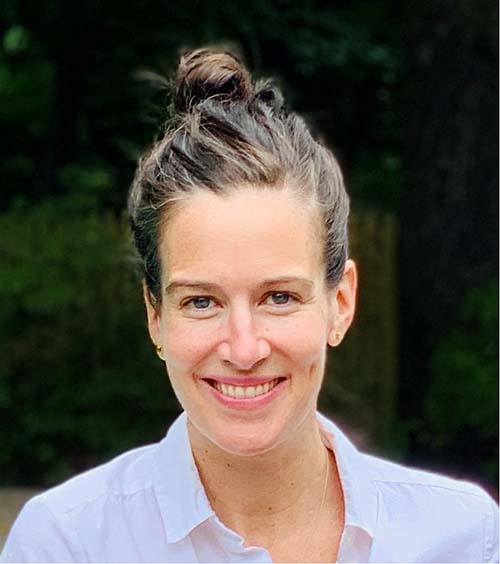
Andrea Blanco Redondo

**[Alexander Szameit]** Since my days as a graduate student, I have been fascinated by the evolution of light in functionalized media, in particular photonic waveguide arrays. In our labs, we fabricate such “photonic circuits” using the femtosecond laser direct-write technology that allows for complex three-dimensional waveguide arrangements to be inscribed into the volume of glass chips. With these technological capabilities at hand, it was a natural progression to the intricate geometries required for implementing topological photonics.

**Figure:**
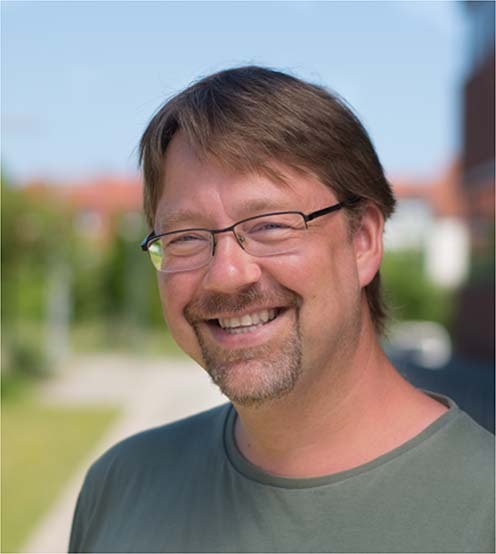
Marco Kirsch

**[Patrice Genevet]** My research activities concern the interaction of light with nanoscale material building blocks, with particular emphasis on nanostructured interfaces, or metasurfaces, for wavefront engineering. Doing so, I have an engineering-oriented approach to the topic of topological photonics in contrast to the original condensed matter physics consideration. During my undergraduate studies, Prof. Pierre Coullet, who is recognized as the pioneer of the singular optical field^1^ taught us fundamental aspects of topological singularities in photonics. I kept working on structured fields during my Ph.D. studies with the investigation of localized optical vortices in semiconductor lasers that added to my fascination for singular optical fields. After our seminal article on “Generalized Snell laws”^2^ with metasurfaces (2011), the field of metasurfaces developed extensively with numerous achievements related to vectorial wavefronts engineering using Pancharatnam-Berry (PB) phase. PB phase is a type of geometric phase that occurs when the polarization is adiabatically modified, and it has been successfully utilized to control amplitude, phase, and polarization of reflected and transmitted fields. During the last 10 years, I kept looking for new physical mechanisms for metasurface designs, and I realized recently the enormous potential of topological photonics for wavefront engineering^3^.

**Figure:**
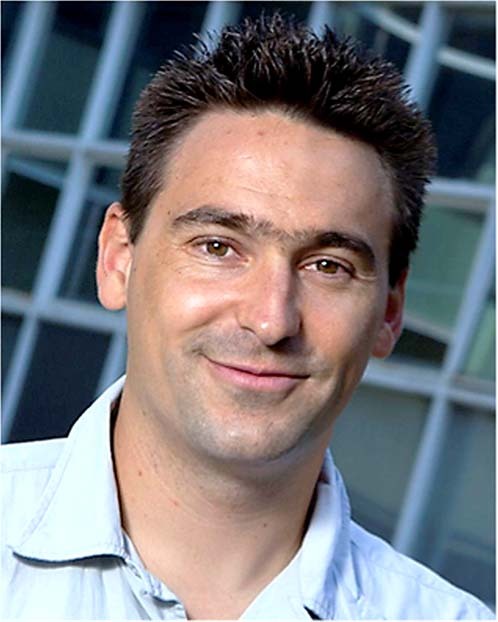
Virgine Genevet

2. Why do we need topological photonics?

**[Bo]** Topological photonics opens a new design space for novel functionalities that can be difficult, or sometimes impossible, to achieve using traditional means.

**[An]** There are many fundamental and practical aspects of why we need to advance in topological photonics. From a fundamental perspective, topological photonics is a very convenient platform to explore topological physics, which is full of possibilities for the discovery of new materials and particles. From the practical side of things, the robustness observed in topological photonics platforms can lead to applications in laser systems, terahertz devices, and fault-tolerant quantum information systems.

**[Al]** Topological photonics is a focus area of research for two reasons. The first is based on plain scientific curiosity: We strive to explore and understand the fundamentals of light evolution, that is, pushing the boundaries of knowledge and overcoming the limitations imposed by the conventional wisdom of what Maxwell’s equations allow light to do. Importantly, pursuing these questions does not only lead to insights into how nature works, but it is also the indispensable basis for novel applications and devices that, down the road, make our life easier and more comfortable. And this is the second reason why we need topological photonics: It provides us with the tools to harness light in ways that were considered to be utterly out of reach until even a few years ago, such as unidirectional three-dimensional routing and switching, and high-performance sensors using long-distance slow light.

**[Pa]** For wavefront engineering you need nothing else than a physical mechanism capable of modifying a complex field $E=A\cdot {e}^{i\phi }$ The latter is characterized by the spatial distribution of its amplitude *A* and its phase $\phi$, respectively varying between [0-1] and [0-2π]. Metamaterials and metasurfaces used so far allow introducing phase shifts to realize various beam shaping configurations. However, limitations remain for applications in terms of spectral range, polarization control, numerical aperture, etc…These limitations basically originate from the difficulty to get $2\pi$ phase shifts for a large range of experimental conditions. From my perspective, topological photonics constitutes an excellent opportunity to complement our meta-optics toolbox. We rely here on innovative effects that are deriving from fundamental topological properties underlying the physics that govern metasurfaces and, in general, physical systems displaying several degrees of freedom. In the vicinity of optical singularities, the amplitude and the phase are rapidly changing. One can thus exploit these rapid changes of the optical response for wavefront engineering. In fact, a variety of optical effects, including Huygens metasurfaces or the reflection near Brewster angle condition, have properties deeply-rooted in topological physics.

3. How would you describe topological photonics and explain its importance to someone who is new to the field?

**[Bo]** In traditional optical systems, when designing an optical component, e.g., an optical waveguide in a photonic chip, the first parameters we focus on are the geometric ones: width, height, shape of cross section. These geometric parameters correspond to their functionalities, such as single-mode operation, tight bending radius, and low group velocity dispersion for nonlinear applications.

In topological photonics, we aim for functionalities that are less thought of or developed. For example, is it possible to engineer a new type of waveguide, which always has a perfect transmission even if with bends and fabrication disorder? Is it possible to design resonances that are less prone to out-of-plane scattering—the current dominant loss mechanism in many optical devices? Is it possible to design an antenna to only radiate towards a single side without the use of a mirror on the other side? Though seemingly impossible at the first sight, these functionalities actually have been demonstrated. The design principles, however, are based on topological phenomena, not geometric parameters.

**[An]** Topological photonics is a relatively new field that leverages the existence of certain global properties of photonic platforms that cannot change easily to provide robust transport of electromagnetic radiation. This is especially important in the context of integrated photonics devices and systems, where even small nanofabrication imperfections can affect the propagation of light, hindering the scalability to the large systems required in many practical applications such as laser arrays, quantum information processing, and neuromorphic computing.

**[Al]** The fascination of topological photonics comes from the fact that topology—an abstract concept that originated in mathematics 150 years ago—is a property of the bulk, that is, from regions of the system far away from its edges. However, the physical impact is felt directly at the edges: So-called topologically protected edge states inherit their incredible degree of stability from the topological characteristics of the bulk. As such, the actual shape of the edges or lattice imperfections cannot impact the propagation of these edge states. In other words, topologically protected edge states have the uncanny ability to find make their way around corners and even through disordered regions, without being subject to any backscattering.

**[Pa]** To discuss the significance of topological photonics beyond its potential of achieving innovative optical applications, as mentioned in question (2), we could take a step back and get a better overview of the foundation of the concepts involved in topological photonics. Topological states refer to a wide class of systems that presents invariant quantities under continuous modification of the parameter space. The concepts developed in this branch of photonics stand on somehow more abstract but pure mathematical considerations that represent an endless source of inspiration. Topological considerations apply to various systems, whether it is a metasurface, a set of coupled microcavities, or a single microcavity pumped below or above the threshold, and the consequences are applicable to a large number of systems that do not seem to be related to each other at the first glance. Topological effects in photonics are interesting as they are providing optical physicists with new states of light, unachievable with conventional optical materials. If photonics is considered today as the technology of the 21st century, or century of light, the development of artificial optical materials, to which topological photonics belongs, is certainly contributing to reinforcing this position.

## Main problems and state-of-the-art

4. In your view and taking your research background into account, how does the existing science and technology contribute toward the development of topological photonics?

**[Bo]** Scientific interest in other topological systems has greatly informed our approach in exploring topology in photonics. Generally speaking, I think of topological photonics as two types: one that focuses on topological defects and one that focuses on topological phases. This distinction is a most superficial one, as there can be a deep connection in between. Interestingly, they both have deep roots in condensed matter physics.

For example, in understanding topological defects in optics/photonics, I benefited from both Gbur’s book^4^ on singular optics as well as Mermin’s 1979 review^5^ paper on the topological theory of defects in ordered media. Due to the similarity between light polarization and liquid crystal orientation, much of the intuition we used in the optical BIC work was built on the understanding of topological defects in nematic liquid crystals—a field seemingly far from photonics.

Meanwhile, topological phases were first studied in the context of condensed matter physics with famous examples ranging from the integer quantum Hall effect to the topological insulators. Due to similarities in eigenvalue problems, these concepts were re-introduced to Maxwell’s equations and later developed into the various topological phases in optical/photonic systems of various dimensions.

Technology-wise, the vast development of nano-fabrication and the general access to it in the academic setting definitely contributed to the development of topological photonics. Improved control over refractive index in photonic structures, both in terms of different materials and small dimensions, has enabled photonic structures that experimentally confirm topological properties.

**[An]** Much of what we know about topological phases of matter can be translated into the photonics realm. For instance, using an analog to the robust edge-propagation in topological insulators in electronic systems, high-slope efficiency topological laser systems and spectrally robust sources of entangled photons have been demonstrated. As another example, topologically protected spatial photonic entanglement has been achieved by using the dimer chain model, initially discovered in polyacetylene chains.

On the other hand, the recent advances in nanofabrication and widespread availability of silicon photonics foundries enable rapid prototyping and testing of new topological photonic structures that may lead to unveiling new physics as well as new applications.

**[Al]** A crucial step in the realization of topological photonic structures was the ability to fabricate complex periodic structures with precision in the sub-micrometer range either in 2D or 3D arrangements. This precision allows for the systematic exploration of the fundamental features of topological light evolution and for new concepts to be put into practice. Along these lines, two of the main technologies responsible for a good deal of the recent advancements in topological photonics have been the femtosecond laser direct-write technology in glass substrates to realize 3D structures as well as electron beam lithography, with which intricate high-contrast planar structures can be fabricated using a variety of material platforms.

**[Pa]** Interestingly, marrying topological properties with metamaterials concepts goes way beyond unidirectional light propagation and topological edge states reported in the literature, and brings topological photonics in uncharted areas. The simple geometrical dependence of the metasurface building blocks and their ease of fabrication with respect to other photonic systems make the metasurface technology a perfect platform to contribute to the development of topological photonics. The control of the phase in the vicinity of singularity can also be combined with conventional metasurface phase addressing mechanisms, such as the Pancharatnam-Berry phase, to achieve disruptive optical designs. Concerning the development of advanced technology, metasurfaces are getting closer and closer to realistic applications, topological metasurfaces and metamaterials could thus enter right away into the realm of industrial applications at optical frequencies.

5. What are the major hurdles to date toward bringing topological photonics out of the lab and into new application areas from your perspective?

**[Bo]** To start with, as the field of photonics and optical technology is well-developed, many practical applications have already been optimized using traditional techniques. The success of topological photonics depends on exceeding the performance of more mature technologies in real-life settings. This involves improving performance beyond the intrinsic limits of traditional techniques in experimental devices and finding new, practical applications that traditional techniques are incapable of performing. Both goals pose major hurdles to the adoption of topological photonics in more practical settings.

Another hurdle can be from the material side. To achieve topological phases, we require various material responses, some of which are simply difficult to have. For example, in optics/photonics, we are still lacking efficient methods to break time-reversal symmetry in the telecommunication/visible regime. This long-standing challenge also propagates to topological photonics because a substantial portion of topological phases require the breaking of time-reversal symmetry. Another example is in achieving Floquet topological phases in driven nonlinear optical systems. For this, we need materials with very strong optical nonlinearities and high damage thresholds, which is also challenging in practice

**[An]** The main hurdle is related to how fundamentally challenging it is to break time-reversal symmetry at optical frequencies, given the weak response of magnetic materials at these frequencies. As a result, none of the currently available topological photonic platforms shows true protection against backscattering at optical frequencies. Another major hurdle—of a more practical nature—is to find ways to integrate photonic lattices of many elements, as required to generate non-trivial topologies, with the current architectures for photonic systems, which are historically based on the use of individual waveguides.

**[Al]** At this point in time, an important milestone towards real-world topological photonics will be the ability to routinely and rapidly synthesize extended three-dimensional photonic structures with feature sizes in the nanometer regime. To the best of my knowledge, currently, no such technology is available. Apart from unlocking higher-dimensional topological features, 3D systems are required to increase the packing density of the optical devices, and the feature size in the nanometer regime is required to address shorter wavelengths in the UV to further decrease the device dimension. Moreover, another fundamental issue in coupled waveguide structures is that commonly, only the energy hopping between the waveguides is topologically protected—while the intrinsic losses of the waveguides and the resulting gradual attenuation along the waveguides are still present. As a result, considerable ground remains to be covered to bring these losses into the range we are accustomed to in optical fibers used for telecommunication.

**[Pa]** Topological metamaterials and metasurfaces have been proposed and discussed in the literature only very recently. From a general point of view, for new concepts to emerge and drive new application domains, the performance of the components, the perspective of innovation, and the compatibility of the technology with existing manufacturing solutions are the key ingredients for the success of the new technology. Topological photonics considered in the framework of metasurfaces and metamaterials is scalable to large volume manufacturing available in semiconductor foundries, but it also shares similar challenges of conventional metasurfaces in terms of efficiency and operational bandwidth. From my perspective and looking at the implementation of topological properties in metasurfaces and metamaterials, the fabrication of low cost and large area optical components with nanometer-scale resolution imposes important manufacturing efforts.

## Important factors to enable practical topological photonics applications

6. Given these hurdles, what steps must be taken to enable the development of topological photonics toward real-world applications?

**[Bo]** To start with, it is important to recognize that scientific discoveries and technological breakthroughs usually do not have well-defined timelines. Therefore, we should always encourage the exploration of novel concepts in topological photonics, while keeping an eye on their possible applications. Once a possible use case is identified, even if seemingly niche, in the beginning, a fair comparison needs to be drawn between topological photonic devices/systems versus existing methods/technologies. Once a promising conclusion is reached and a proof-of-concept demonstration is achieved in the lab, it is probably beneficial to have industrial partners or startup companies involved to further develop or even commercialize such devices and/or systems. Overall, developing a successful eco-system involving universities, funding agencies, start-up companies, and industry would be an important step in bringing topological photonics into the real world.

**[An]** One research approach towards backscatter-free propagation in topological photonics is to work on avenues to break time-reversal frequency at optical frequencies. Some of these avenues include the use of dynamical modulation of the coupling strengths between lattice elements and the use of acoustic pumping. It has also been theoretically suggested that truly backscatter-free propagation could be achieved in parity, time-reversal, and duality invariant platforms of nonperiodic continuous media. Working on realistic implementations of these and other ideas is one way of solving this fundamental challenge for topological photonics.

Further, to efficiently integrate topological lattices into real applications, a new holistic approach to photonic system design is required, in which the use of the lattice is an advantage and not a burden. For instance, laser systems involving many lasing elements, rather than just one waveguide or resonator, can in principle achieve much higher powers. As another example, the use of waveguides or resonator lattices in quantum information processing opens the possibility of having many spatial modes and leveraging these for multimode entanglement. Combining these advantages, inherent to the use of lattices, with the robustness given by topology, can lead to real-world applications.

**[Al]** We are in urgent need of a novel 3D lithography technology for fabricating large-scale structures with nanometer resolution in materials that are transparent in UV. With such a next-generation platform at hand, tailored effective media could be realized without relying on discrete waveguide arrangements and their comparably high intrinsic losses. This approach would therefore play a major role in the implementation of existing and future topological concepts with reasonable efficiency and compact footprint.

**[Pa]** To advance the development of topological metamaterials, concrete realizations that are improving the performances of classical optical components have to be considered. While it is clear that metasurfaces and metalenses possess attractive properties in terms of optical functionalities, weight reduction, and accessible proof-of-concept fabrication processes, it is not yet clear which part of the market could topological metasurfaces be taking in the future. The applicability of topological concepts with other metasurface phase addressing mechanisms indicates that topological photonics will find applications in the areas covered by metasurfaces and metamaterials in general.

## Possible application domains and useful technological advances

7. What potential is there for topological photonics to become a disruptive technology?

**[Bo]** Applying concepts and design principles in topological photonics to appropriate optical/photonic platforms is probably the best approach.

One such example is the photonic integrated circuits—a fast-developing platform that is revolutionizing communication, sensing, augmented reality/Virtual reality, and other technologies. This platform is also naturally close to topological photonics, as a significant portion of the proof-of-concept demonstrations in topological photonics were already implemented in the integrated form.

Another possible platform is fiber optics, which has been seminal in long-haul communication, fiber sensing, and generating light sources. It can be very fruitful to introduce topological protection to the fiber systems.

**[An]** The potential to leverage the robust electromagnetic propagation of topological photonics is enormous. However, for topological photonics to become a disruptive technology it needs to be able to solve the actual problems of integrated photonics systems. Achieving truly backscatter-free light propagation would guarantee a disruption, enabling large-scale photonic integration. But even before reaching this ambitious goal, there are some other technologies – such as quantum lattices and laser arrays - that could be disrupted by leveraging topological robustness to slight differences in the sizes or the couplings of the elements in an array.

**[Al]** Already, topological photonics has challenged and changed many of our views about the very nature of light evolution in integrated photonic devices, and allows for functionalities that seemed impossible before. Although most proposals are still on the drawing board or in the stage of fundamental research, I have no doubt that topological photonic devices will play a major role in future technologies that will advance our modern global society.

**[Pa]** For any technology to become a disruptive technology, it is necessary to propose nontrivial capabilities that are not accessible with any other means, which is the case of topological photonics. Looking at the latest results in topological photonics, not only those related to metamaterials and metasurfaces, gap-less frequency edge-states that display unidirectionality and robust waveguide modes, topologically immune to local disorder, have the potential to become a disruptive technology. These have interesting applications in particular for robust propagation and filtering in photonic integrated circuits, lasers, and optical isolators. In practice, even small fabrication imperfections, sometimes smaller than the wavelength, are detrimental to photonic components. Topologically-protected transport using protected edge states could find applications. The flexibility in designing artificial optical systems to any wavelength of interest, and their compatibility with topological properties occurring 1,2 and 3 dimensional structured are certainly great advantages of topological photonic technology.

## New functionalities with topology and future outlook

8. What are potential application areas for future topological photonics research? Connected to this, in your view, what further steps should the research community take toward the development of practical topological photonics applications?

**[Bo]** On the one hand I believe that photonic integrated circuits have much potential to deploy topological photonics systems and I think this could be a great application domain. On the other hand, a major part is to discuss with people knowing the needs of the market and the state of the art. A fair comparison, in terms of the pros and cons, between topological photonic approaches and existing technology needs to be made. Even if the conclusion is promising, often significant additional engineering work needs to be done before a practical topological photonics application can be delivered. It´s difficult to put a timeline on this development, considering, since scientific discoveries and technological breakthroughs tend to happen unexpectedly. That said, commercialization has its own time requirements so once a breakthrough occurs, there will always be some lag between the experimental demonstration of a practical application in a research lab and its widespread adoption.

**[An]** I find the application of topological photonics to quantum technologies the most exciting. Quantum photonic states are particularly fragile and susceptible to fabrication imperfections, this makes it particularly interesting to apply topological concepts to robustly generate, transport, and interfere such states. Other interesting domains of applications are lasers and sensors.

However, for the most part of the last decade, the community focused on creating and understanding photonic analogs of topological matter systems. Only in the last few years, we have witnessed the first research attempts to develop real topological photonics applications such as lasers, quantum systems, and sensors

The research community should start a more concerted effort to understand the actual requirements of individual applications and focus its efforts on those applications that can truly benefit from the types and degree of robustness that topological photonics can offer at the moment.

**[Al]** I foresee two main fields where topological photonic devices will play a major role. One is optical interconnects in hybrid quantum computers, where the actual computations will be executed by superconducting circuits or trapped ions, and robust information transfer between these processors is enabled by entangled photons populating topological states. The second is enhanced sensing applications using slow light, where, by virtue of topological protection, slow light may cover genuinely macroscopic distances.

As I mentioned before, I think that a key prerequisite for practical applications of topological states is the development of a 3D lithography technology for large-scale structures with nanometer resolution.

Furthermore, an important course of action is to familiarize the applied-sciences community, including the R&D departments of small and medium-sized enterprises (SMEs) as well as larger enterprises with the concept of topological photonics and the technological capabilities it may enable. Similarly, another immediate step is increased patenting of ideas that might be feasible as technological applications. If my hopes come true, the aforementioned 3D lithography technology will be become available in 5 years, allowing these ideas to serve as a springboard for the ensuing topological photonics revolution.

Last but not least, the development of devices that are relevant for industrial applications requires a lot of (both) time and resources. Hence, applied research in topological photonics requires substantially more funding compared to what is devoted to this field to date.

**[Pa]** Topological photonics has the potential to drive new applications in the area of quantum optics, nanophotonics, fiber optics, and manipulation of structured light. Topological metasurfaces, among other metamaterials, will have a significant impact in the design and conception of compact and functional future components. I also expect innovative light-matter interaction using topological edge modes and topological cavities, thereby bridging the gap between topological concepts as applied to photonics and those as applied to condensed matter. In this direction, the success of implementing topological photonics within the next 5 years requires focused R&D aiming at the realization of high technology readiness levels (TRL~5-7) demonstrations. The latter are needed to analyze in detail the device performances and to benchmark topological photonics systems with competing PIC technologies. Efforts in achieving large-scale design optimization and large-area fabrication will be needed. In particular, these issues have to be brought up to foundries so as to adapt and adjust the nanofabrication processes and to reduce the manufacturing cost of the components. Methods and solutions to properly and reliably integrate these demonstrations in functional systems, i.e. a display or a sensor, are also needed. Anyway, it is always hard to determine the time it would take for emerging technology to be used in practical applications as it depends not only on the advancement of the technology but also on its readiness level and on the interest of the industrials to implement innovative solutions in their product development and perspectives. I expect at least 3 to 5 years of continuous efforts in research and development (R&D) before seeing practical systems relying on topological photonics.

9. Lastly, do you have any suggestions for how researchers in relevant fields can work together more effectively in what is likely to become a multidisciplinary field?

**[Bo]** In any collaboration, good communication is critical. In interdisciplinary contexts, it is easy to get lost in jargon, so forming common vocabulary, or at least clarifying differences or similarities between vocabularies, is incredibly helpful. To this end, it is important to start with a common ground that is concrete and comprehensible to researchers from different fields. This can be a specific physical phenomenon, a mathematical definition, a type of material response, or the performance figure of merit for a device or a system. In this concrete context, we can convey the different design principles and new possible functionalities more easily across subject boundaries.

Another aspect is the willingness to stretch what research looks like in a certain field and take creative risks. Since multidisciplinary work requires input from different fields, the research itself will require stretching outside what any one field is comfortable with.

**[An]** To this day, topological photonics remains a somehow obscure topic for large part of the photonics community. This is because topological photonics stems from earlier discoveries on condensed matter physics and little effort has been made on making it approachable to people with other scientific and engineering backgrounds. Researchers in topological photonics must make an effort to effectively communicate the advantages and the challenges of their research to other communities, especially to the quantum and the integrated photonics communities. In turn, input from these communities is crucial to ensure that the efforts in topological photonics are directed toward solving real challenges rather than simply looking for problems for their solution.

**[Al]** Experts have to find ways to present their results in a more comprehensible fashion. While this is a truism across fundamental science, topology is a notoriously complex topic, such that it needs significant effort to efficiently convey the key messages to a broader audience, ideally while steering clear of doughnuts and coffee cups in the one extreme, and page-filling equations on the other.

**[Pa]** Promoting the technology to industry could lead to focused university-industry partnerships or joint research centers that could consolidate topological photonics as a multidisciplinary field. Lobbying of the technology by leaders and recognized scientists who are able to identify challenges and opportunities of topological photonics could trigger high-level political commitment and could mobilize extensive resources. The development of topological photonics could be associated with large-scale initiatives aiming at accelerating seriously the development of disruptive photonic solutions. The consolidation of, for example, a European Flagship program on disruptive photonics with artificial materials that would comprehend advanced photonic surfaces, topological photonics, and space-time metamaterials can totally transform the photonics industry, accelerate the innovation and generate strong international cooperation. The future of photonics with artificial materials is bright, extremely exciting and moving forward one-way only with topological photonics.

*The interview was conducted by Cristiano Matricardi, Cephas Small and Lina Persechini*.
